# Anatomical approach for left parahisian ventricular arrhythmia ablation to reduce atrioventricular block risk

**DOI:** 10.1093/ehjcr/ytaf549

**Published:** 2025-10-30

**Authors:** Federico Vannini, Francesco Raffaele Spera, Luigi Salerno, Emanuele Barbato

**Affiliations:** Department of Clinical and Molecular Medicine, Sapienza University, Rome, Italy; Cardiology Department, Sant’Andrea University Hospital, Italy; Department of Clinical and Molecular Medicine, Sapienza University, Rome, Italy; Cardiology Department, Sant’Andrea University Hospital, Italy; Department of Clinical and Molecular Medicine, Sapienza University, Rome, Italy; Cardiology Department, Sant’Andrea University Hospital, Italy; Department of Clinical and Molecular Medicine, Sapienza University, Rome, Italy; Cardiology Department, Sant’Andrea University Hospital, Italy

## Case description

We present the case of a 73-year-old man who experienced palpitations and presyncope in 2021. A 24 h Holter ECG showed frequent monomorphic premature ventricular contractions (PVCs) with a 15% burden, refractory to beta-blockers, and flecainide. Amiodarone was avoided due to age and toxicity risk.

Baseline 12-lead ECG showed narrow QRS complexes, qR in V1, prominent R waves in V3–V6, and intermediate axis (lead II positive, lead III negative), suggesting a left parahisian origin^1^. Echocardiography revealed preserved left ventricular ejection fraction (LVEF 60%) and no structural disease.

**Figure 1.**
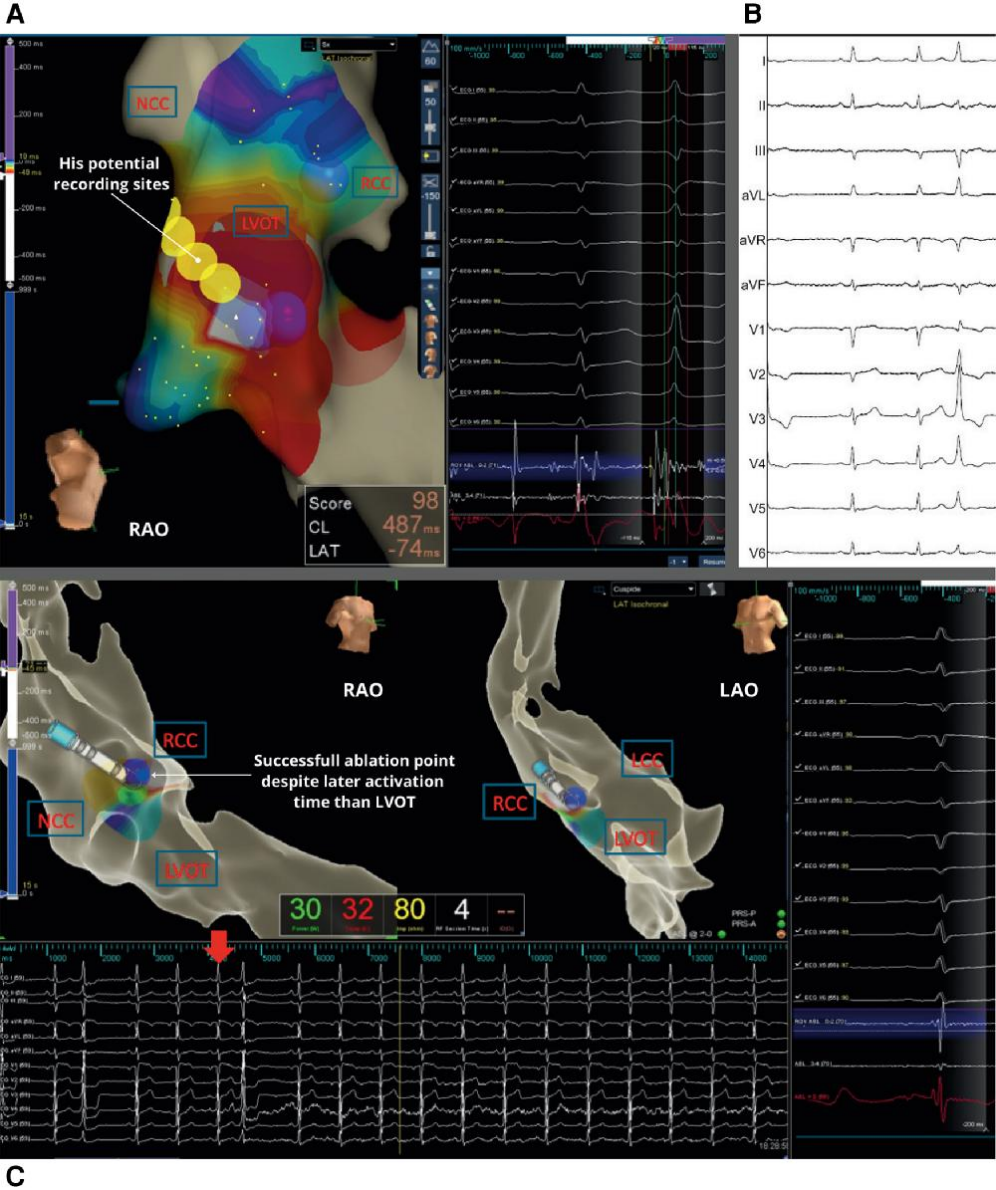
(*A*) Left ventricular outflow tract 3D electroanatomical activation map of left parahisian PVC. On the left side of the figure, electroanatomical reconstruction of the aortic cusps and left ventricular outflow tract. Circles indicate regions with His potential recordings; spheres mark the site where ablation was performed. In the middle, surface ECG and intracardiac EGM recordings at 100 mm/s speed during sinus rhythm (first beat) and PVC (second beat) show the earliest signal at the parahisian region 74 ms before the onset of the QRS complex. From top to bottom, the traces show surface ECG leads, intracardiac ablation distal and proximal bipolar (Abl D-2, Abl 3-4), and distal unipolar (Abl D+) EGMs. (*B*) Baseline 12-lead surface electrocardiogram of unifocal left parahisian PVC: narrow QRS complexes due to an early engagement of the His Purkinje network, qR in V1, prominent R waves in V3–V6, and intermediate axis (lead II positive, lead III negative). (*C*) Successful ablation point in the right coronary cusp of left parahisian PVC. On the left side an electroanatomical reconstruction of the aortic cusps and left ventricular outflow tract in RAO (left) and LAO (right) projection with activation mapping at the RCC indicating the sequence of activation from the earliest to the latest activated site. Spheres show the site of successful ablation after 4 s of radiofrequency at 30 W. At the bottom the 12-lead ECG during ablation, with the arrow indicating the time when ablation started. As shown, no further PVCs were recorded after a few seconds of ablation. On the right side of the figure surface ECG and intracardiac EGM recordings at 100 mm/s speed during PVC show the earliest signal on the right coronary cusp, only 42 ms before the onset of QRS complex without an optimal unipolar signal. From top to bottom, the traces show surface ECG leads I, II, III, aVR, aVL, aVF, and V1–V6, intracardiac ablation distal and proximal bipolar (Abl D-2, Abl 3-4), and distal unipolar (Abl D+) EGMs.

Given the atypical PVC morphology, initial management followed guidelines recommending antiarrhythmic therapy, but persistent symptoms led to catheter ablation. ECG findings (R wave in V1 and early precordial transition) suggested a left-sided origin, so mapping was performed via a retrograde aortic approach using EnSite X™ 3D mapping system (*Figure 1*).

Activation map from the aortic cusps showed earliest ventricular activation at the right coronary cusp (RCC), 42 ms before QRS onset, but with a small ‘r’ in the unipolar signal, indicating proximity but not the true origin. Mapping of the left ventricular outflow tract (LVOT) showed earlier activation, 74 ms before QRS onset with optimal unipolar ‘QS’ morphology, but a sharp His potential during sinus rhythm indicated close proximity to the conduction system. Two radiofrequency applications (RF) at 40 W were delivered 1 cm away from this area, but proved ineffective. Given the risk of AV block, further ablation was avoided. Mapping of the right ventricular septum revealed activation 31 ms before QRS onset, again close to the His-bundle and with less favourable timing.

Considering activation timing and safety, the RCC was selected. Absence of a His potential indicated safer distance from the conduction system while preserving activation timing. This approach is anatomically supported, as the RCC overlies the membranous septum, which contains the His-bundle. RF at 30 W eliminated PVCs within 4 s. PQ and HV intervals (HV 50 ms) were continuously monitored and remained unchanged after ablation.

Risks, including AV block and pacemaker implantation, were discussed before the procedure.

This case shows that RCC ablation can be a safe and effective option in selected patients with left parahisian PVCs—defined here as the earliest activation with QS unipolar morphology in the LVOT where a His potential was recorded. This approach should be individualized based on anatomy and detailed mapping findings.

## Data Availability

All available data relevant to this case report have been included within the manuscript.

## Notes

**Consent:** Written informed consent for publication of this case report and any accompanying images was obtained from the patient in accordance with COPE guidelines.

**Funding**: None declared.
